# Rare Sensory Ataxic Variant of Guillain-Barre Syndrome: A Case Report

**DOI:** 10.7759/cureus.40920

**Published:** 2023-06-25

**Authors:** Pavithera Packiyarajah, K.T Sundaresan, M.S.M Nusair

**Affiliations:** 1 Internal Medicine, Teaching Hospital Batticaloa, Batticaloa, LKA; 2 Department of Clinical Sciences, Eastern University of Sri Lanka, Batticaloa, LKA

**Keywords:** difficulty in diagnosis, importance of diagnosis, presentation, rare variant, guillain-barre syndrome (gbs)

## Abstract

The sensory ataxic variant of Guillain-Barre syndrome (GBS) is a rare subtype, with limited case reports available. We present the case of a previously healthy 26-year-old female university student who presented with bilateral foot numbness and unsteady gait for five days, without limb weakness. There were no signs of infection or recent history suggestive of infection. Examination revealed reduced pain and light touch sensation, as well as proprioception impairment in the bilateral distal lower limb, accompanied by an ataxic gait. Bilateral upper and lower limb power was normal. Cerebrospinal fluid (CSF) studies showed albuminocytological dissociation, while nerve conduction studies indicated unrecordable sensory responses with normal motor responses. Through a comprehensive evaluation of history, examination, and investigations, other potential differential diagnoses were excluded. Then the patient was diagnosed with a sensory ataxic variant of Guillain-Barre syndrome and treated with intravenous immunoglobulin (IVIG). Over time, the patient demonstrated gradual improvement and was able to resume her university studies four months after discharge.

## Introduction

The initial identification of Guillain barre syndrome (GBS) depicted it as a neurological condition distinguished by ascending muscular weakness, deficient reflexes, and comparatively mild sensory manifestation. However, over time, it has become evident that GBS is a clinically and electro-physiologically diverse condition that encompasses various variants. One such rare variant is the sensory ataxic form of Guillain-Barre syndrome, which was first discussed in the 1950s. The sensory ataxic variant of GBS is a result of autoimmune damage to the sensory nerves with an underlying mechanism of molecular mimicry. The incidence rate of the sensory ataxic variant is relatively low and additionally, the condition may be underdiagnosed or underreported because of its variable presentation. The identification of sensory ataxic GBS poses challenges, but prompt diagnosis is crucial to prevent unnecessary complications and ensure favorable outcomes. To the best of our knowledge there have been no reported cases of the sensory ataxic variant of Guillain-Barre syndrome in Sri Lanka and we would like to report this rare variant of GBS.

## Case presentation

A previously healthy 26-year-old university student presented with a five-day history of bilateral foot numbness and unsteady gait. She had been well until five days prior to admission. The patient initially experienced numbness in her right foot, followed by numbness in the left foot within a day. Simultaneously, she lost her balance and was unable to walk, but she did not exhibit any upper or lower limb weakness. All of the above symptoms progressed rapidly within five days and presented to the hospital. She denied facial asymmetry, slurred speech, swallowing difficulties, incontinence, or visual problems. Autonomic symptoms were absent.

There were no accompanying symptoms of headache, fever, vomiting, constitutional symptoms, or recent history of infection. The patient had no history of exposure to toxins or illicit drug abuse, and there was no evidence of trauma. Additionally, there were no suggestive features of connective tissue diseases, and her family history was unremarkable. She didn’t have a coronavirus disease 2019 (COVID-19) infection and had not received COVID-19 vaccination in the past or any other vaccinations in the last month.

Upon examination, the patient was alert and oriented to time, place, and person. Vital signs were within normal limits, including a pulse rate of 88 beats per minute, blood pressure of 130/80 mmHg, respiratory rate of 18 breaths per minute, and oxygen saturation of 98% on room air. She exhibited adequate respiratory effort with a good cough reflex.

Cranial nerve examination revealed no abnormalities, including the absence of nystagmus, ptosis, or ophthalmoplegia. The tone and power of bilateral upper and lower limbs were normal, but she demonstrated global areflexia. The plantar reflexes were downgoing. Sensory examination revealed symmetric impairment of pain, light touch, and proprioception in the distal part of both lower limbs just above the ankle, without a discernible sensory level. She had truncal ataxia. Romberg's sign was positive. Cerebellar examinations were normal. Abdominal, cardiovascular, and respiratory examinations yielded normal findings, with no signs of dysautonomia.

**Figure 1:**
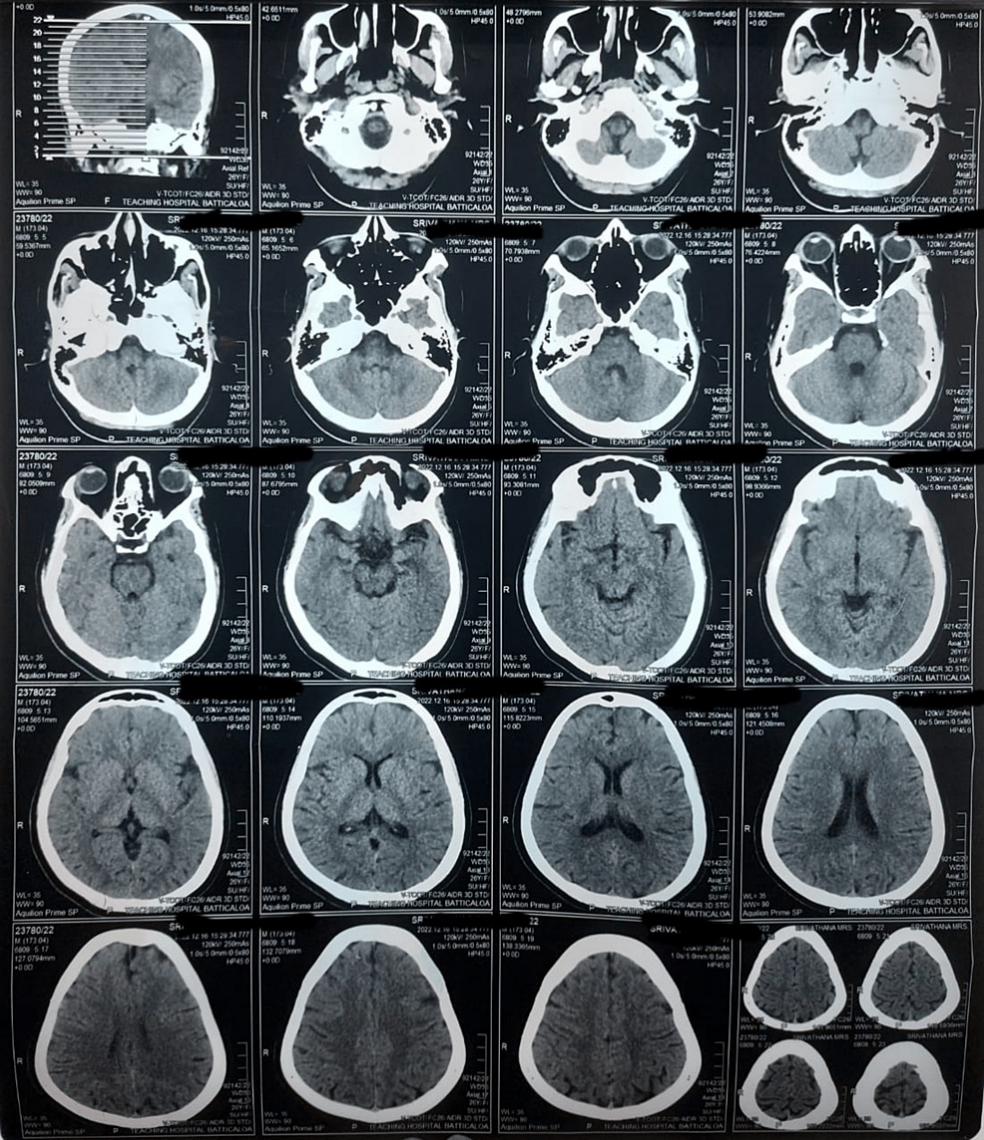
Non-contrast CT (NCCT) Brain of the patient- Normal NCCT Brain

Baseline investigations, including full blood count, liver function tests, renal function tests, and inflammatory markers, were all normal (Table 1). Non-contrast CT scan of the brain (Figure 1) and MRI of the brain showed no abnormalities. The nerve conduction study revealed unrecordable sensory responses, while motor responses were normal, suggesting a sensory variant of Guillain-Barre syndrome or ganglionopathy.

**Table 1:** Table of relevant investigations of the patient on admission and on discharge PLT: platelets, S.Cr: serum creatine, ALT: alanine aminotransferase, AST: aspartate aminotransferase, ALP: alkaline phosphatase, GGT: gamma-glutamyl transferase, CRP: c-reactive protein, ESR: erythrocyte sedimentation rate, TSH: thyroid stimulating hormone, NCCT: non-contrast CT

Investigation	Normal values with units	On the Date of admission	On the Date of discharge
WBC	4-11 ×10^3^/UL	9.2	7.6
HB	12-16g/dl	11.8	12.4
PLT	150-450×10^3^/ul	349	369
Na+	136-145mmol/l	139	142
K+	3.5-5.1mmol/l	4.2	4.1
S.Cr	62-115µmol/L	38	46
AST	15-37U/L	19	17
ALT	12-78U/L	30	26
ALP	44-116U/L	66	
Total protein	64-82g/L	76	
Albumin	34-50g/L	38	
GGT	15-85U/L	19	
T.Bilirubin	3.4-17µmol/L	12	
CRP	0-5	2.2	3
ESR		48	44
TSH	0.35-4.94Mu/lL	1.74	
ECG		normal	
2D echo		normal	
CXR		normal	
USS Abdomen		normal	
S.Ca^2+^	2.1-2.5mmol/l	2.2	
Mg^2+^	0.7-1.0mmol/l	0.8	
NCCT Brain		normal	

A contrast-enhanced CT scan of the chest, abdomen, and pelvis was performed to rule out a primary tumor with paraneoplastic manifestation, which showed normal results. Cerebrospinal fluid (CSF) analysis demonstrated albumino cytologic dissociation (Table 2). Testing for GT1a and GQ1b antibodies, which would help confirm the diagnosis, could not be conducted due to financial constraints.

**Table 2:** Cerebro spinal fluid (CSF) report of this patient

Appearance	clear	
Protein	62mg/dl	(15-45mg/dl)
Polymorphs	nil	
Lymphocytes	4	
Red cells	8	
CSF Glucose	63 mg/dl	Random blood sugar 122mg/dl

Based on the patient's clinical presentation, the exclusion of other alternative diagnoses, and supportive investigations, a diagnosis of a sensory ataxic variant of Guillain-Barre syndrome was made. The patient received intravenous immunoglobulin (IVIG) at a dosage of 0.4 mg/kg/day for five days, along with supportive care including physiotherapy and balance training exercises.

Following treatment, the patient didn’t show immediate improvement but demonstrated a gradual improvement. On admission, her GBS disability score was 4, which decreased to 2 after three months. Four months later, she was able to resume her studies at the university.

## Discussion

Guillain-Barre syndrome (GBS) is an acute polyradiculoneuropathy characterized by areflexic motor symptoms, with or without sensory symptoms. Within the spectrum of GBS, there are several atypical variants, including Miller-Fisher syndrome, pharyngeal-cervical-brachial variant, acute bulbar palsy, pandysautonomia, Bickerstaff brainstem encephalitis and the sensory ataxic variant, which is a rare subtype.

These atypical variants are often associated with antibodies against GQ1b, GT1a, and GD1b, which are distributed in the peripheral nerve myelin sheets. Molecular mimicry plays a role in the production of these antibodies [1]. While there are few case reports of the sensory ataxic variant of GBS, the diagnostic criteria for sensory loss and areflexic variants of GBS were proposed in 1981 and updated in 2001 [2]. These criteria include acute symmetrical sensory loss, a peak in symptoms at four weeks, absent tendon reflexes, normal muscle power, evidence from electrophysiological examination, a single-phase course, exclusion of other neurological conditions, absence of a family history of similar presentations, and albuminocytologic dissociation [3].

In our patient, the predominance of sensory symptoms without weakness, along with areflexia and supportive findings from the nerve conduction study and cerebrospinal fluid analysis, support the diagnosis. Other potential causes of the same presentation, such as multiple sclerosis, cerebellar ataxia, and paraneoplastic manifestations, were excluded through history, examination, and investigations.

Sensory neuropathy (ganglionopathy) is a major differential diagnosis of the sensory ataxic variant, characterized by prominent pain and dysesthesia, typically involving the face and scalp [4,5]. Unlike GBS, sensory neuropathy is subacute or chronic.

Nerve conduction study findings in sensory GBS often show temporal dispersion, prolonged sensory nerve action potential (SNAP) latency, slow conduction velocity, and delayed or absent F waves, with or without motor involvement [6].

GBS is typically a post-infectious and immune-mediated disease, with most cases following upper respiratory tract or gastrointestinal infections. Common pathogens associated with GBS include Campylobacter jejuni, Cytomegalovirus, Epstein-Barr virus, and Mycoplasma pneumoniae, with Campylobacter jejuni being the most frequently identified agent [7,8]. There have also been significant cases of GBS following COVID-19 infections or COVID-19 vaccination [9,10]. GBS affects both sexes equally, with a higher incidence in adults compared to children.

The GBS disability score is used to assess the functional status of patients [11]. Management of GBS involves monitoring for cardiac and pulmonary dysfunction, prevention of pulmonary embolism, and immunotherapy with IVIG or plasma exchange. IVIG initiated within two weeks of symptom onset is effective, as is plasma exchange [12].

The overall prognosis for GBS is generally good, with nearly 85% of survivors having good functional recovery. Early diagnosis and treatment are essential for reducing mortality and improving outcomes.

## Conclusions

Early diagnosis and treatment with IVIG are crucial for improving outcomes in sensory ataxic Guillain-Barre syndrome (GBS). This case emphasizes the importance of timely intervention, as the patient demonstrated gradual improvement and a decrease in GBS disability score. Healthcare professionals should consider GBS as a differential diagnosis in patients presenting with sensory symptoms and ataxia, even in the absence of limb weakness. Sharing case reports contributes to the existing literature and underscores the significance of recognizing and managing this rare GBS variant for better patient outcomes.
